# A machine learning approach for the factorization of psychometric data with application to the Delis Kaplan Executive Function System

**DOI:** 10.1038/s41598-021-96342-3

**Published:** 2021-08-19

**Authors:** J. A. Camilleri, S. B. Eickhoff, S. Weis, J. Chen, J. Amunts, A. Sotiras, S. Genon

**Affiliations:** 1 Institute of Neuroscience and Medicine (INM-7 Brain and Behaviour), Forschungszentrum Jülich, Jülich, Germany; 2 Institute of Systems Neuroscience, Heinrich-Heine University, Düsseldorf, Germany; 3 Department of Psychology and Behavioral Sciences, Zhejiang University, Hangzhou, China; 4 Mallinckrodt Institute of Radiology, Institute for Informatics, Washington University in Saint Louis, Saint Louis, USA

**Keywords:** Human behaviour, Cognitive neuroscience

## Abstract

While a replicability crisis has shaken psychological sciences, the replicability of multivariate approaches for psychometric data factorization has received little attention. In particular, Exploratory Factor Analysis (EFA) is frequently promoted as the gold standard in psychological sciences. However, the application of EFA to executive functioning, a core concept in psychology and cognitive neuroscience, has led to divergent conceptual models. This heterogeneity severely limits the generalizability and replicability of findings. To tackle this issue, in this study, we propose to capitalize on a machine learning approach, OPNMF (Orthonormal Projective Non-Negative Factorization), and leverage internal cross-validation to promote generalizability to an independent dataset. We examined its application on the scores of 334 adults at the Delis–Kaplan Executive Function System (D-KEFS), while comparing to standard EFA and Principal Component Analysis (PCA). We further evaluated the replicability of the derived factorization across specific gender and age subsamples. Overall, OPNMF and PCA both converge towards a two-factor model as the best data-fit model. The derived factorization suggests a division between *low-level* and *high-level* executive functioning measures, a model further supported in subsamples. In contrast, EFA, highlighted a five-factor model which reflects the segregation of the D-KEFS battery into its main tasks while still clustering higher-level tasks together. However, this model was poorly supported in the subsamples. Thus, the parsimonious two-factors model revealed by OPNMF encompasses the more complex factorization yielded by EFA while enjoying higher generalizability. Hence, OPNMF provides a conceptually meaningful, technically robust, and generalizable factorization for psychometric tools.

## Introduction

As of late, research in psychological and medical sciences has been subject to a replication crisis^1–4^ that has infiltrated many disciplines interested in human behavior including differential psychology and cognitive neuroscience^2,5–7^. This crisis stems from the finding that a vast number of research results are difficult or impossible to replicate^8^. Several contributing factors have been pointed out and possible solutions have been proposed. Among the contributing factors, the limited sample size and the flexibility in the choice of analysis appear to play an important role^9–12^. Specific choices in the sample selection, measure of interest, and the criteria for significance, together with specific criteria for evaluating the relevance or validity of the analysis’ outcomes are examples of factors that directly influence the final findings and conclusions of any study. This problem has been fully acknowledged and extensively discussed in the context of hypothesis-driven studies (i.e., testing a specific psychological effect), and potential solutions for the problem have been suggested. Pre-registration of confirmatory hypotheses has been recommended to limit a-posteriori choices driven by questionable practices such as p-hacking and data-fishing^13^. However, these practices are more difficult to implement in the case of exploratory studies of human behavior, where the analysis is data-driven rather than hypothesis-driven. This applies to the search for latent structure in psychological data capitalizing on multivariate approaches. Actually, the replicability issue has been rarely raised in this domain, despite the influence of the choice of analysis on the findings has been often discussed^14,15^.

A popular exploratory method widely used in psychological research is exploratory factor analysis (EFA), which has been introduced in the field by Spearman^16^. It aims to reduce a number of observed variables to fewer unobserved factors in order to identify a hidden structure in the data and to facilitate interpretability^14^. In a conceptual or theoretical perspective, these structures are used as constructs in sophisticated models describing different aspects of human behavior. The established models and structures are then considered as a ground theory on which following studies can build to further characterize human behavior. For example, studies have built on derived factorial models of executive functioning to establish relationships with other aspects of human behavior^17^, to examine genetic influences^18^, or to propose neural substrates^19^ of this cognitive function. In that context, an exploratory factor analysis is generally used to identify latent structure in a set of behavioral variables, such as a test battery, and the derived structure then serves as a model which is usually a-priori imposed on a new dataset using a confirmatory factorial analysis^12^. Nevertheless, as noted by Treiblmaier and Filzmoser^14^, many factor solutions can be derived from one correlation matrix and the final solution represents just one of many possible choices. Analyses methods, such as the EFA, involve a number of choices that require the researcher to make crucial decisions that have a substantial impact on the results and subsequent interpretation^20–23^. Such decisions include the number of factors to retain and the criteria used to select this, the type of rotation applied, and the interpretation of the resulting factor solution^24^. These are choices, that, in addition to the data collection aspects such as sample size and test battery, can have an influence on any type of study. Consequently, the lack of replicability of factorizations in the literature has been reported in a number of fields. For instance, one can point out the diverse and inconsistent factor solutions proposed for psychiatric scales^25^; personality scores^26^, and executive functioning^27–31^. In this context, and considering the broader framework of the replication crisis in psychological research, it appears necessary to question the utility and generalizability (i.e., the external validity) of exploratory approaches to identify latent structure in psychological tools. Traditionally, Principal Component Analysis (PCA) has also been used for the investigation of the latent structure of behavioral data. To date, the literature is not in agreement as to which method is most appropriate in the context of behavioral data. Many authors argue against the use of PCA mainly because this is considered to be solely a data reduction method and not a true method of factor analysis in a psychological sciences perspective^32–35^. However, other authors disagree^36,37^. Generally, the main point of debate concerns the perspective in which the factorization is applied. As aforementioned, EFA specifically aims to identify hypothetical constructs (also referred to as factors, dimensions, latent variables, synthetic variables or internal attributes). In the behavioral sciences, these latent dimensions are assumed to be unobservable characteristics of people. Accordingly, the factors derived from an EFA are expected to have a theoretical validity. In contrast, PCA aims to provide a summary representation of the original variables into components, without having the specific aim to reflect theoretical constructs. Given their different aims, EFA and PCA have different ways of conceptualizing sources of variance in measured variables. EFA assumes that factors are not perfectly reflected by the measured variables, and thus distinguishes between variance in measures due to the common factors and variance due to unique factors. On the other hand, PCA does not make such a distinction and the resulting components contain a combination of common and unique variance^38^. Considering this distinction further implies that EFA factors are assumed to reflect latent constructs, and thus should not be expected to vary across subsamples. In contrast, from a data-science perspective, PCA and data reduction approaches in general, could be expected to provide different representations depending on the datasets by extracting a simplified representation of the data. Given these differences between the two approaches, the choice of one approach over the other can influence the result, perpetuating the problem of replicability in the identification of latent structures.

Executive functioning is one of the most studied psychological concepts in psychology and is continuously examined in cognitive neuroscience. Executive functioning refers to processes central to coordinated, goal-directed behavior and is thought to play a major role in a wide range of different psychiatric and neurological diseases^39^. However, despite its significance, the true nature of executive abilities remains rather elusive. One of the main reasons for this is that executive functioning is not a single process but rather a “macro-construct” encompassing various aspects of mental functioning^40^. Moreover, the lack of a clear formal definition of executive functioning is also due to the nature of the aspects that constitute it, the relationship among these and their contribution to the overall concept^41^. As a result, there is a constant interest in the study of the structure of executive functioning and its relationship with other traits and behaviors^17^. Throughout the years, several neuropsychological tests have been designed to capture and measure different executive abilities. However, the measurement of executive functioning poses several challenges^41–44^ including the fact that executive functioning tests tend to be inherently impure^29^. Executive functioning operates on other cognitive processes, and thus any score derived from an executive functioning task will unavoidably include systematic variance that can be attributed to non-executive functioning processes associated with that specific task context^42,44^. This latter issue is referred to as the task impurity problem and is addressed by using factor analytical techniques. These map the shared variance between tests of executive functioning to a set of latent variables, providing a cleaner estimate of these higher-order cognitive abilities than the individual tests^42,45^. Consequently, numerous studies have investigated the latent structure of executive functioning using different factorization methods and executive functioning batteries. However, the different studies have resulted in diverse findings and conceptual models^27–31^. The long-term study of factors, or components, of executive functioning is thus particularly illustrative of the plurality of latent structures that can be derived from factorization methods in psychological research for a particular concept.

In the clinic, the most popular way of assessing executive functioning is by using test batteries that evaluate the diverse higher-order abilities through multiple tests^44^. One such test battery that has become increasingly common in clinical practice, as well as in research, is the Delis–Kaplan Executive Function System^46^. The D-KEFS is one of the first normed set of tests developed specifically to assess executive functioning. It consists of nine tests comprising traditional and newly developed tests covering a wide spectrum of verbal and non-verbal executive functions, which are all designed to be stand-alone instruments that can be administered individually or together with other D-KEFS tests. Past studies have used different methods to attempt to evaluate the latent structure of this particular battery, identifying some evidence of diverse latent factors explaining performance on individual tests^17,45,47,48^. In summary, the D-KEFS represents a widely used psychological tool with applications in clinical settings, but for which different factorizations could be proposed in the healthy population.

Considering the heterogeneous factorization results in the literature of executive functioning and psychology in general, generalizability should be a crucial criterion of validity in order to reach a conceptual consensus in psychological sciences. However, as can be seen in the study of executive functioning, a plethora of models exists. In the context of a replicability crisis in psychological sciences, the heterogeneity of models is particularly problematic. The use of different models that examine different aspects of interindividual variability prevents comparison and integration across studies. However, practically evaluating generalizability is hard due to lack of data (and lack of funding support for replicability evaluation). This is particularly the case for factorization analyses, which require large sample sizes for each evaluation. Nevertheless, internal cross-validation can be used to give insight on how the model will generalize to unseen data that are not used for model derivation. As a common approach in the machine learning field, cross-validation consists of the partitioning of a dataset into subsets. The analysis is then performed on one subset (the training set) and validated on the other subset (the test set) across multiple runs with different training and test sets.

In recent years, the increased use of machine learning approaches has emphasized the use of internal cross-validation to increase robustness and to estimate generalizability to an independent dataset. This has led to the popularization of novel methods, which can also be used as factorization techniques, thus offering a novel perspective for behavioral sciences. While these novel approaches are commonly perceived as lacking interpretability and validity when compared to classical statistical approaches, some methods have been developed with the purpose of increasing these aspects by adding additional constraints. One such method, the OPNMF (or Orthonormal Projective Non-Negative Matrix Factorization), provides a relatively higher interpretability as compared to more traditional methods, such as the classic NMF. OPNMF was recently used to identify a robust and generalizable factor structure of Positive and Negative Syndrome Scale (PANSS) data from participants with schizophrenia^25^. The new factor-structure was moreover shown to more reliably relate to specific brain functions than the original PANSS subscales, demonstrating the usefulness of this OPNMF approach^49^. This technique could hence significantly contribute to the definition of robust factorization of psychological variables, in particular for widely used psychological tools, such as standard neuropsychological batteries, socio-affective questionnaires and clinical scales.

The motivation of this study was two-fold. Firstly, given the importance of generalizability in the identification of latent structures, one main goal of the present study was to compare the factorization obtained when using a machine learning approach (OPNMF) with a cross-validation scheme, with the factorization derived from more traditional approaches that tend to lack the generalizability aspect, in particular EFA, but also PCA. Furthermore, a second motivation of this study was to better understand the nature of EF and the tasks commonly used to investigate it. To this end, we capitalized on a large open access dataset of healthy adult scores of the D-KEFS provided by the Enhanced Nathan Kline Institute – Rockland Sample. This dataset is heterogenous in covering the whole adult life span, providing a good gender balance and including participants from the whole population (including different ethnicities), thus making it optimal for this study in which generalizability is central. EFA and PCA were here performed by using standard statistical techniques as implemented in open access statistical tools such as JASP^50^. Furthermore, the choice of the optimal number of factors or components for these traditional approaches was based on recent guidelines in the field, while the choice of the optimal number of components for OPNMF was based on standard criteria assessing not only the quality of the data representation, but also its generalizability. Finally, to further evaluate the quality of the different factorizations, we examine the stability or generalizability across age and gender subsamples.

## Methods

### Sample and measures

The current study used age-corrected scaled D-KEFS scores of 334 adults (18–85 years old; mean age = 46; 101 males) obtained from the Enhanced Nathan Kline Institute—Rockland Sample (eNKI)^51^. Written informed consent was obtained from all participants. The local ethics committee of the Heinrich-Heine University in Düsseldorf, Germany approved analysis of the data and all methods were carried out in accordance with relevant guidelines and regulations. The main variables of the analyses included 17 D-KEFS Total Achievement Scores (Table 1), which reflect global achievement scores on the 9 tests included in the D-KEFS battery and broadly reflect traditional measures of executive functioning^46^. Only participants that had scores for all 17 variables were included in the study resulting in the exclusion of 385 participants from the original eNKI dataset. Additional information regarding the education level and occupation of the participants can be found in the supplementary material. This study used five different (sub) groups: (1) the full dataset including 334 adults; (2) a subset of the data including only males (n = 101); (3) a subset of the data including only females (n = 233); (4) a subset of the data only including subjects aged over 50 (n = 144); and (5) a subset of the data only including subjects aged 50 or under (n = 220).

**Table 1:** Description summary of all variables included in the study.

Test	Variable	Variable description	Measure
Trail making test	Number-Letter Switching	Requires examinees to switch back and forth between connecting numbers and letters in sequence	Completion time [s]
Verbal fluency	Letter Fluency	Requires examinees to say as many words as possible starting with a specific letter in 60 s	Sum of correct responses
	Category Fluency	Requires examinees to say as many words belonging to a specific semantic category in 60 s	Sum of correct responses
	Category Switching	Requires examinees to switch between two specific categories in 60 s	Sum of correct responses
Design fluency	Design Fluency—Filled dots	Measures the examinee’s ability to draw as many different designs as possible in 60 s	Total number of correct designs
	Design Inhibition—Empty Dots only	Measures the examinee's ability to draw as many different designs as possible in 60 s while making sure that certain responses are inhibited	Total number of correct designs
	Design Switching	Measures the examinee's ability to draw as many different designs as possible in 60 s while requiring participants to engage in cognitive shifting	Total number of correct designs
Color word interference	CWI—Inhibition	Requires examinee to inhibit reading the words in order to name the dissonant ink colors in which the word is printed	Completion time [s]
	CWI—Switching	Requires examinee to switch back and forth between naming the dissonant ink color and reading the word	Completion time [s]
Sorting test	Confirmed Sorts	Participants are required to sort cards into two groups according to as many different categorization rules or concepts as possible	Total number of correct sorts
	Free Sorting Description	Participants are required to describe the concepts they used to generate each sort	Total number of correct descriptions
	Sort Recognition	Participants are required to identify the correct categorization rule or concept used to sort cards that have been sorted by the examiner	Total number of correct recognitions
Twenty questions test	Initial Abstraction Score	Examinee is shown pictures of common objects and the task is to ask the fewest number of yes/no questions possible to identify the object chosen by the examiner	Minimum number of objects eliminated by first question
	20 Questions—Total Achievement Score		Sum of weighted achievement scores across all items
Word context test	Word Context—Total Achievement Score	Examinee attempts to discover the meaning of a made-up word on the basis of its use in five clue sentences	Consecutively correct items
Tower test	Tower Test—Total Achievement Score	Examinee is required to move disks varying in size across three pegs to build tower in the fewest number of moves possible to match the target tower while following certain rules	Sum of achievement scores (summed up for all items)
Proverb test	Proverb Test—Total Achievement Score	Proverbs are read individually to the examinee who is required to interpret them orally without assistance or cues	Sum of achievement scores (summed up for all items)

The D-KEFS battery offers a wide range of tests that tap into many of the established constructs of executive functioning. The D-KEFS battery includes the following tests: (a) **Trail Making Test**, which aims at assessing attention, resistance to distraction and cognitive flexibility; (b) **Verbal Fluency Test**, which assesses the ability of generating words fluently from overlearned concepts and thus reflects efficient organization of such concepts; (c) **Design Fluency Test**, which is a non-verbal version of the Verbal Fluency Test and assesses the ability of quickly generating designs; (d) **Color-Word Interference Test**, which taps into inhibition and cognitive flexibility by assessing the ability to inhibit an overlearned verbal response in order to generate a conflicting response; (e) **Sorting Test**, aims at measuring multiple components of concept-formation and problem-solving abilities; (f) **Twenty Questions Test**, which assesses the ability to formulate abstract questions and to come up with problem-solving strategies; (g**) Word Context Test**, assesses skills such as deductive reasoning, information integration, hypothesis testing, and flexibility of thinking; (h) **Tower Test**, which assesses spatial planning and rule learning; and (i) **the Proverb Test**, which tests abstraction abilities. All variables included in the present study are presented in Table 1. All variables were examined for outliers and visually inspected for inappropriate distribution. Frequency distributions for each of the 17 EF variables used in the analyses can be found in the supplementary material.

### Factorization of D-KEFS scores using OPNMF

NMF is a factorization method that enables the decomposition of a given matrix into two non-negative matrices: (1) a basis matrix with columns representing the resulting latent factors and (2) a factor-loading matrix representing the loading coefficients. The two resulting matrices together should approximate the original data matrix. NMF and its variants have been widely used in various recent biomedical studies including metagene discovery^52^, classification of cancer subtypes^53,54^, identification of structural brain networks^55^, and identification of dimensions of schizophrenia symptoms^25^. Such applications of NMF and its variants have shown that such methods do not require the input data to be normally distributed. One such variant, the OPNMF, has in fact been shown to derive stable and generalizable factor solutions for data with skewed distributions^25,49^. The present study aims at discovering the latent structure of executive functioning by applying this promising method to D-KEFS performance scores. In order to achieve this in an interpretable fashion, the present study adopted a specific variant of NMF, the OPNMF, which adds additional constraints to the algorithm in an effort to promote sparsity and hence improved interpretability to the results^25,55,56^.

The OPNMF algorithm was first applied to D-KEFS total achievement scores coming from the whole sample, with the number of factors ranging from 2 to 9. Additionally, the algorithm was applied to the subsets of the dataset that were split by gender and age. The optimal number of factors, and hence the most robust, stable, and generalizable factor model, was identified by using cross-validation in 10,000 split-half analyses^25^. Considering the different sizes of the sub-samples, the cross-validation scheme that was used (i.e., partitioning the dataset into subsets and then performing the analysis on the training set and validating it on the test set across multiple runs with different training and test sets), ensured the robustness of all analyses, including the ones using smaller subsets of the dataset, in a more direct way than classical power and its use in classical statistics. Specifically, the eNKI sample was split into two halves, and OPNMF was performed on each split sample to derive the basis matrix. Subsequently, each item was assigned to a specific factor based on its largest coefficient within the basis matrix. The adjusted Rand index^57^, and variation of information^58^ were then employed to assess the stability of item-to-factor assignments between the basis matrices derived from the two split samples. Although OPNMF generates almost clustering-like structure, it allows small contributions from multiple items to specific factors. Hence we further evaluated the stability of the whole entries by comparing the two basis matrices as assessed by the concordance index^59^. For the adjusted Rand-index and concordance index, a higher value indicates better stability across splits, while for the variation of information metric, better stability corresponds to lower values. Generalizability was assessed by quantifying out-of-sample reconstruction error by projecting the data of one split sample onto the basis matrix from the other split sample. A lower increase in out-of-sample error compared with within-sample reconstruction error indicates better generalizability^25^. All analyses were run using Matlab R2018a with customized codes, which are available upon request.

### PCA and EFA

Data from each of the five different matrices was additionally subjected to exploratory factor analysis (EFA) and principal component analysis (PCA). In both analyses, loading matrices were rotated using promax oblique rotation as currently suggested in the field^15^. An oblique rotation (which allows correlation between the factors) was chosen because of an a priori expectation that higher order factors would reflect a coherent domain of executive functioning, as suggested by the goals of the D-KEFS^46^. Furthermore, previous studies showed that executive functioning tasks tend to be correlated^42,60–62^, hence justifying the use of oblique rotation. In both EFA and PCA, the optimal number of factors/components was determined by using two different methods: the Scree test^63^ and eigenvalue Monte Carlo simulation approach^64^, ( i.e., parallel analysis) The Scree Test has been traditionally used for the selection of number of factors and involves plotting the eigenvalues in descending order of their magnitude and determining where they level off to ultimately select the number of meaningful factors that capture a substantial amount of variance in the data^65^. On the other hand, parallel analysis simulates a set of random data with the same number of variables and participants as the real data from which eigenvalues are computed. The eigenvalues extracted from real data that exceed those extracted from random data then indicate the number of factors to retain^15^. This method formally tests the probability that a factor is due to chance and hence minimizes the over-identification of factors based on sampling error^66^. It is thus superior to the reliance upon eigenvalue scores generated by factor analytic processes alone. Parallel Analysis has also been shown to perform well when determining the threshold for significant components, variable loadings, and analytical statistics when decomposing a correlation matrix^67^. Finally, for the reader’s information, we also reported here a typical goodness-of-fit measure in EFA, the Tucker-Lewis Index (TLI). TLI reflects the ratio of the model chi-square and a null-model chi-square. In the null-model, the measured variables are uncorrelated (thus there are no latent variables), consequently the null-model has usually a large chi-square (i.e., a poor fit). TLI values express the goodness-of-fit of the found model relative to the null-model and usually range between 0 and 1. As a rule of thumb, a value > 0.95 indicates a good fit , a value > 0.90 indicates an acceptable fit for and a value < 0.90 indicates a poor fit^68^.

## Results

### Optimal number of factors across different factorization approaches and subsamples

**Figure 1:**
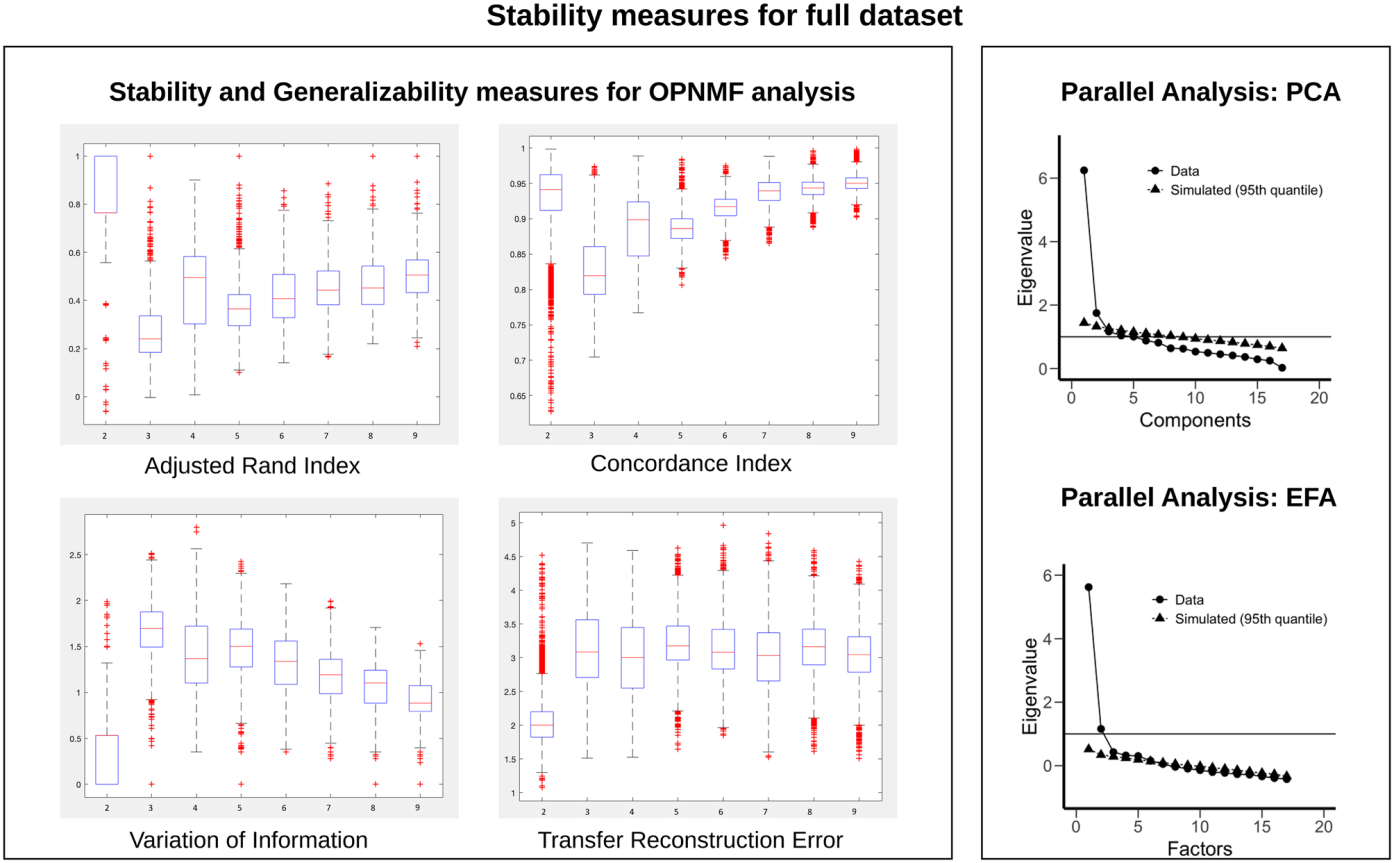
Stability measures for full dataset. Left panel shows plots for each of the stability measures used to identify the most robust factor solution for the OPNMF analysis. The right panel shows plots for the parallel analyses used to identify the most robust component/factor solutions in the PCA and EFA analyses.

Based on results of the stability measures (Fig. 1), the OPNMF analysis on the full dataset indicated a two-factor model as the optimal solution. The adjusted Rand index, variation of information and concordance index between the basis matrices, all indicated the two-factor solution to be the most stable. The transfer reconstruction error indicated that the 2-factor solution was the most generalizable. Stability measures for the OPNMF analyses that were carried out on subsets of the data split by gender and age showed a similar pattern to the ones resulting from the full dataset, thus suggesting a two-factor model for each of the subsets of the data. Both the Scree plot and the Parallel analysis carried out for PCA also indicated that the optimal solution consisted of a 2-factor model for the full dataset analysis. This 2-factor model was consistent for most PCA analyses performed on the data subsets when looking at both selection indices with the exception of the male subset whose scree-plot indicated a 4-factor solution. Consistently, in the case of the EFA analyses, the Scree plot indicated a 2-factor model for the full dataset analysis (TLI = 0.732) as well as for all the analyses performed on the data subsets (male: TLI = 0.670; female: TLI = 0.761; older adults: TLI = 0.745; younger adults: TLI = 0.699, all suggesting a poor fit). However, the parallel analyses results yielded more heterogenous findings. EFA parallel analyses results carried out on the full dataset suggested a 5-factor solution (TLI = 0.931 suggesting an acceptable fit). When the full dataset was split by gender, the EFA analyses results suggested a 3-factor solution for the male subjects only dataset (TLI = 0.837 suggesting a poor fit) and a 5-factor solution for the female subjects only dataset (TLI = 0.906 suggesting an acceptable fit). When the full dataset was split by age, the EFA analyses results suggested a 4-factor solution for both older (TLI = 0.894 suggesting a marginally acceptable fit) and younger (TLI = 0.732 suggesting a poor fit) age groups. Given the previous literature showing that Parallel Analysis performs well (Franklin et al., 1995), as well as the TLI indices that have resulted from our analyses, the Parallel analysis was chosen to be the index of choice. Consequently, the results reported below use the factor-model that was indicated by parallel analyses for both EFA and PCA. Figures showing the stability measures for each of the subsets of the data can be found in the supplementary material.

### Factorization structure across different factorization approaches and subsamples

**Figure 2:**
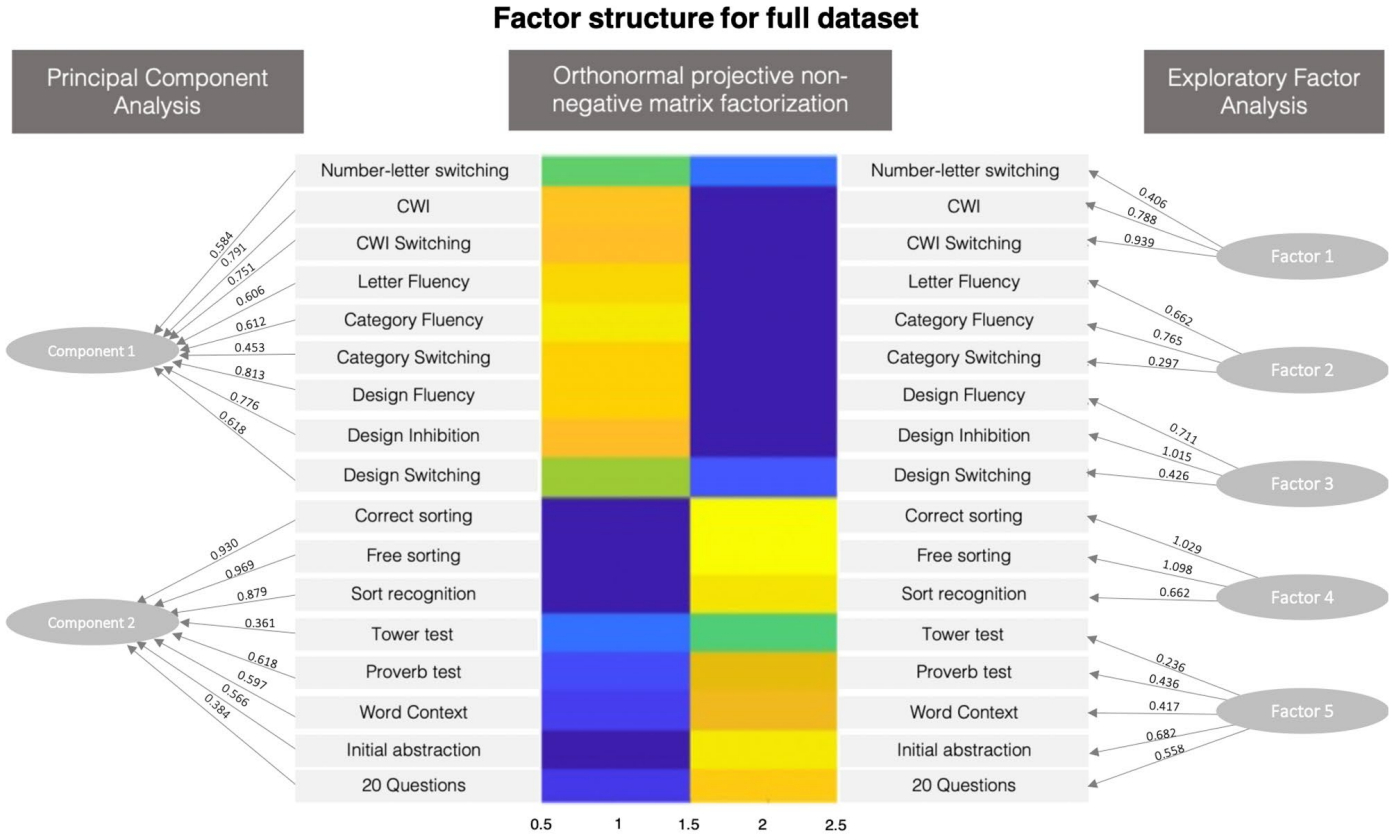
Factor structure and factor loadings resulting from the PCA, EFA and OPNMF analyses for the full data set. Figures show strongest loadings for each variable.

In the case of the OPNMF carried out on the full dataset, the resulting two factor solution consisted of one factor strongly loading on Color-Word Interference (CWI), Verbal Fluency and Design Fluency scores and moderately loading on switching components of the Design Fluency Test and the Trail Making Test. The second factor featured strong loadings on the Sorting Test, Proverbs Test, Word Context Test and the 20 Questions Test and a weaker loading on the Tower test (Fig. 2). This pattern was mostly consistent throughout the different subsamples of the data that were split by gender and age, with some minor exceptions. In the case of males only dataset, both switching components of the Verbal Fluency Test and the Trail Making Test showed weak loadings onto the first factor, while the switching component of the Design Fluency Test showed a stronger loading. In the case of females only dataset, the Word Context Test showed weak loadings onto the second factor together with the Tower Test. When the full dataset was split by age, the Tower Test, Proverb Test and Word Context Test all showed weak loadings onto the second factor in the dataset consisting of older adults, while the 20 Questions Test loaded weakly onto the second factor together with the Tower Test in young adults. Noticeably, all subsamples showed the same tests loading onto each of the two factors.

The PCA analyses resulted in component models that showed patterns that were strikingly similar to the OPNMF models for the full dataset as well as for each of the subsets. The component model resulting from the analysis of the full data set resulted in a two-factor solution that consisted of one factor strongly loading on CWI scores and Design Fluency scores and moderately loading on Verbal Fluency Scores and the Trail Making Test. The second factor featured strong loadings from the Sorting Test, moderate loadings from the Proverbs Test and Word Context Test and a weaker loading for the Tower test and the 20 Questions Test (Fig. 2). This pattern was repeated when the PCA analyses were carried out on subsets of female sand younger adults. When the PCA analysis was run on a subset that included only males, the factor solution consisted of one factor strongly loading on CWI scores and Design Fluency scores, moderately loading on Verbal Fluency Scores and the Trail Making Test and weakly loading on the Tower Test and the 20 Question Test. The second factor featured strong loadings from the Sorting Test, moderate loadings from the Proverbs Test, Word Context Test and weaker loading on the 20 Questions Test. The factor solution for the males only dataset consisted of one factor strongly loading on CWI scores and Design Fluency scores, moderately loading on Verbal Fluency Scores and the Trail Making Test and weakly loading on the Tower Test and the Word Context Test. The second factor featured strong loadings from the Sorting Test, moderate loadings from the Proverbs Test and the 20 Questions Test.

The EFA analyses resulted in a more heterogenous picture. The EFA analysis of the full dataset resulted in a five-factor solution consisting of one factor including scores from the Sorting Test; one factor that included scores from the CWI Test and the TMT test; one factor including scores from the Design Fluency Test; one factor including scores from the Proverbs Test, Word Context Test, 20 Questions Test and Tower Test; and another factor including scores from the Verbal Fluency Test. The EFA results for the males only dataset showed a three-factor solution with one factor including scores from the Sorting Test; one factor including scores from the Tower Test, Word Context Test and 20 Questions Test and the switching component of the Verbal Fluency Test; and one factor including the rest of the scores from the Verbal Fluency Test; the Trail Making Test, the Color-Word Interference Test, and the Design Fluency Test. In the females only dataset, the resulting factor structure consisted of a five-factor solution with one factor including scores from the Sorting Test; one factor including scores from the Verbal Fluency Test; one factor including two scores from the Design Fluency Test; one factor including the Trail Making Test, scores from the Color-Word Interference Test, the switching component of the Design Fluency Test, the Tower Test and the Word Context Test; and a final factor including scores from the Proverb Test and 20 Questions Test. When the full dataset was split by age, the EFA resulted in a four-factor solution in both subsets. In the case of the older adults dataset, the resulting factor structure consisted of one factor including scores from the Sorting Test; one factor including scores from the Verbal Fluency Test, the Color-Word Interference Test, the Trail Making Test and the Word Context Test; one factor including scores from the Design Fluency Test and the Tower Test; and a final factor including scores from the Proverb Test and 20 Questions Test. In the case of the younger adults dataset, results showed one factor including scores from the Verbal Fluency Test; one factor including scores from the Design Fluency Test; one factor including scores from the Color-Word Interference Test, and the Trail Making Test; and a factor grouping scores from the Sorting Test, Tower Test, Proverb Test, Word Context and 20 Questions Test. Result figures for each of the subsets can be found in the supplementary material. Importantly, all EFA and PCA analyses were replicated using another open access statistical software, Jamovi^69^ (version 1.2, https://www.jamovi.org), and resulted in virtually identical results.

## Discussion

Although the field of psychology has acknowledged and discussed the existence of a replicability crisis extensively, this issue has received less attention in the context of multivariate approaches for psychometric data factorization. This has resulted in heterogenous factorization results for several constructs in psychology, including executive functioning. Given the importance of replicability and generalizability in the identification of latent structures, the main goal of the present study was to compare the factorization obtained when using a machine learning approach (OPNMF) with a cross-validation scheme with the factorization derived from more traditional approaches, in particular EFA, but also PCA, in the D-KEFS. These latter approaches were performed as typically implemented in standard statistical software and following current guidelines, which usually do not include generalizability evaluation. In addition to the evaluation of factorization approaches, this study provides further insight into the specific nature of the D-KEFS and hence also contributes more generally to the understanding of executive functioning. The following paragraphs start with a discussion of the results of the EFA analysis with regards to previous literature together with EFA theoretical background. We then discuss the convergent results obtained when using OPNMF and PCA from a methodological point of view and also with regards to previous literature on executive functioning and the related evaluation tools. Finally, we discuss the resulting two-factor solution in the context of a parsimonious and robust representation of executive functioning for various applications.

### EFA analysis

Using traditional EFA analysis, our investigations of the factorization across subsamples first indicate that the optimal solution can vary across subsamples, hence suggesting that the generalizability of the factor solution derived by an EFA analysis can be relatively limited. Overall, in the whole dataset, a five-factor solution appeared to be the best model fit. This result suggests a segregation that reflects the structure of the D-KEFS battery with the Sorting, Design Fluency and Verbal Fluency Tests each being assigned to their own factors, while tasks that require a certain level of abstraction and problem-solving abilities were grouped together in one factor. Thus, overall, the factorial analysis was here strongly influenced by the specific structure of the test battery that was used. It is noteworthy that this finding is somewhat contradictory with the core assumption behind EFA that states that EFA reveals unobservable latent variables reflecting meaningful psychological constructs. A similar, albeit not identical structure is seen when performing an EFA on females only. In the case of males, results suggested a three-factor solution, while in both younger and older adults the EFA indicated a four-factor solution. The evaluation of the theoretical validity of the factorization derived here by the EFA in a psychological science perspective is complicated by the fact that the literature reports a multitude of different factor models, including various factor solutions, all using different methods of factorization, datasets and test batteries. In particular, similar exploratory studies that used EFA have also resulted in heterogenous factor solutions ranging from one^70^ to six factors^71^.

One model of executive functioning that has acquired a significant amount of empirical support is the three-factor model by Miyake et al.^42^. This influential study uses a confirmatory analysis approach as opposed to the exploratory approach established in the present study, and factorizes executive functioning into shifting, inhibition and updating. Shifting refers to the ability to switch between operations and perform new operations while being faced with interference^42^. Inhibition requires the ability to purposefully control automatic or dominant responses^42^. Finally, the updating factor represents tasks that require the monitoring and evaluating of new information and, if necessary, the updating of information in working memory for the successful completion of the task at hand^42^. Interestingly, the EFA findings of the present study do not overlap with the shifting, inhibition and updating factors suggested by Miyake et al.^42^. However, it is noteworthy that the three-factor model presented by Miyake and colleagues^42^ was based on a limited set of tasks and did not include an exhaustive list of executive functions. Specifically, Miyake’s study^42^ and others^61,72^, have focused mostly on tasks that require simpler cognitive abilities, and thus tend to not include tasks that tap into more complex abilities, such as problem-solving, abstraction and strategic thinking. On the other hand, the D-KEFS battery, which was used in the present study, offers a wide range of tests that tap into many of the established constructs of executive functioning, including more complex abilities, such as abstraction, reasoning, and problem solving^46,73^. Unsurprisingly, the specific set of tasks used will heavily impact the resulting factor model. The literature does include studies that have attempted to factorize D-KEFS measures using both confirmatory and exploratory approaches. Hence, Karr and colleagues^45^ used confirmatory factor analysis, which led them to the conclusion that the D-KEFS taps into three EF factors, namely, inhibition, shifting and fluency. However, this study chose not to include tasks that tap into more complex abilities (i.e., Twenty Questions, Word Context, and Proverb Tests) in the input variables. On the other hand, Latzman and colleagues^17^ used EFA to factorize D-KEFS measures and reported a three-factor model comprising Conceptual Flexibility, Monitoring and Inhibition, which was likened to the Miyake model by the authors.

A number of subsequent studies have supported the three factors of shifting, inhibition and updating presented by Miyake et al.^42^ by reporting similar three factor solutions from a series of confirmatory factor analyses of diverse cognitive tasks^45,61,72,74^. Other similar confirmatory approaches have resulted in different factor solutions depending on the age group that was investigated^75–79^. To further understand the heterogeneity of findings reported in the literature and the divergence between the results of the EFA in the current study and previous conceptualization, it is important to note here that there is a fundamental difference between confirmatory and exploratory approaches in terms of their use to identify latent factors. Confirmatory approaches, such as Confirmatory Factor Analyses, use knowledge of the theory of the construct and previous empirical findings to test a hypothesis that has been postulated a priori. Therefore, the aim of this approach is to verify a specific factor structure of a set of observed variables*.* This approach will hence provide an evaluation that is in alignment with current research^45^, however will be undeniably impacted by the initial research hypothesis used. On the other hand, exploratory approaches identify the underlying factor structure of a set of variables without the need of establishing an a priori hypothesis. The latter, thus, allows for the deeper understanding of a construct in an exploratory fashion. In other words, confirmatory approaches can be considered as “hypothesis-driven” approaches to some extent, while exploratory approaches can be considered as “data-driven” approaches. Differences in results when comparing confirmatory and exploratory factor analyses are therefore not surprising.

### OPNMF and PCA

While suggesting a different factorization than EFA, PCA and OPNMF together converge toward a similar 2-component model. It is noteworthy that this convergence was observed despite the fact that the choice of optimal factor solution was based on different criteria within and between approaches including the part of variance explained, data representation quality and stability evaluations. PCA and OPNMF factorization methods here resulted in one factor that designated loadings to Color-Word Interference scores, Verbal Fluency, Design Fluency Test and the Trail Making Test. The second factor featured strong loadings from the Sorting Test, Proverbs Test, Word Context Test and the 20 Questions Test and a weaker loading for the Tower test. These results seem to indicate a division between tasks that require monitoring and task-switching, and more complex tasks that require concept formation, abstraction, and problem-solving. Specifically, tasks that require a certain level of abstraction, strategic thinking and problem-solving abilities, such as the Sorting Test, Twenty Questions Test, Word Context Test, Tower Test, and the Proverb Test, were all grouped into one factor. On the other hand, tasks that require less complex abilities were grouped in another factor. The latter factor includes tests that tap into abilities such as monitoring, fluency, cognitive flexibility, and inhibition. Hence, in contrast to previous results, our results obtained from the OPNMF and PCA analyses suggest a stable and robust two factor model indicating a division between *Simple* and *Complex* (or *low-* vs *high-level*) executive functioning tasks. While previous factorization findings of executive functioning do not seem to support our findings indicating a split between *Simple* and *Complex* tasks, it has been previously shown that people suffering from executive functioning impairment, such as in the case of patients with mild cognitive impairment, tend to exhibit selective rather than global impairment with some studies showing a separation between impairment on simple versus more complex tasks^80–82^. The idea of simple versus complex is also reflected in neurobiological literature in which a separation of tasks between the dorsolateral prefrontal cortex and the ventrolateral prefrontal cortex^83^ has been suggested. The former has been implicated in the context of more complex aspects of executive functioning although not all evidence supports this^83^. The notion of separation of tasks based on complexity is also in line with the proposed hierarchical organization of the frontal cortex^84^. When taking a deeper look at the individual measures that were included in the present study, it becomes apparent that there is a noteworthy difference between the different measurement approaches used and the subsequent processes that they could be eliciting. Specifically, while some of the variables are measures of accuracy (e.g., correct number of items), others rely more heavily on time pressure and processing speed (e.g., reaction time and completion time). This difference in measurement approaches seems to be reflected in the resulting dichotomy between *Simple* and *Complex* tasks. In fact, whereas the *Complex* tasks quite clearly emphasize accuracy, the *Simple* tasks appear to be more overtly driven by the element of time. The number-letter switching task, CWI and CWI switching are all direct measures of time while the other variables that have been grouped together with the *Simple* factor are measures of fluency which arguably also involves an aspect of time pressure since its measurement is related to time efficiency when recalling items. Additionally, although the factor labelled as *Complex* in the present study includes measures that tap into abilities such as reasoning, abstraction, problem-solving, and strategic thinking, this factor also includes measures coming from the Sorting Test. The D-KEFS Sorting Test and tests with a similar procedure, such as the Wisconsin Card Sorting Test^85^, have been traditionally associated with the *Shifting* or *Conceptual Flexibility* factor^17,42,45,74^. This association appears to be appropriate since the Sorting Test and its variants require participants to shift from previous sorting rules to new rules to achieve a greater number of accurate sorts. However, the Sorting Test also taps into more abstract problem-solving strategies that go beyond simple shifting. This complexity of the Sorting Test is reflected by the results of the present study. Thus, the factorization derived from PCA and OPNMF appears parsimonious and meaningful from a psychological construct standpoint. This study hence demonstrated that the application of machine learning approaches to psychometric data can provide interpretable outcomes in a psychological science perspective. It should be noted here as well that OPNMF further promotes out-of-sample generalizability by evaluating reconstruction error in a left out set across multiple runs, which is a crucial aspect considering the replication issues in psychological sciences.

Despite the apparent divergence of factorization results between EFA on the one hand and OPNMF and PCA on the other hand, it should be noted that the results of our EFA analyses provide a higher factor model that reflects the segregation of tasks that was used in the D-KEFS battery while still assigning a single factor to tasks that require abstraction and problem-solving skills. Hence, the parsimonious two factor model can also be seen as encompassing the more complex factorization yielded by EFA. The results of the present study thus suggest that the OPNMF and PCA results provide a robust and stable two factor solution that separates tasks that require monitoring and task-switching from more complex tasks that require concept formation, abstraction, and problem-solving. Considering all the points discussed above, together with the fact that both methods converged towards one robust model, we suggest that our results may reflect a robust factor model that applies across a wide age range and across different factorization methods. Given the uncertainty and diverse findings of the factorial structure of executive functioning in the literature, this model offers a more scientifically parsimonious model from both technical and conceptual standpoints. From a technical standpoint, the approach established in the present study (i.e., that of reaching a consensus among different technical variations) is the most reasonable to our knowledge since it is commonly known that different approaches can result in different factor solutions. From a conceptual standpoint, the 2 factor solution presented in this study results in a scientifically parsimonious model since the differentiation between *Simple* and *Complex* is better at reflecting consensual real-world concepts than models with a higher number of factors. Considering these scientific qualities, the robust and parsimonious two-factor model that emerged from this study should be of higher practical utility for characterizing inter-individual variability in executive functioning performance at both the biological level (such as genetic and brain subtrates) and the environnmental level (external factors).

### Summary and conclusion

In addition to demonstrating the advantages of a machine learning approach for the factorization of psychometric data in a replicability perspective, this study also provides a robust model of factorization of the D-KEFS. The derived factorization suggests a division between *low-level* and *high-level* executive functioning measures, a model further supported in subsamples. In contrast, EFA, highlighted a five-factor model as the better fit to the overall cohort, but which was poorly supported in the subsamples. This five-factor factorization reflects the segregation of the D-KEFS battery into its main tasks while still clustering higher-level tasks together. Thus, the parsimonious two-factors model revealed by OPNMF underlies the more complex factorization yielded by EFA while enjoying higher generalizability. Hence the application of OPNMF to psychometric data in the present study provides conceptually meaningful, technically robust and generalizable factorization for psychometric tools.

## Supplementary Information

Supplementary Figure 1.

Supplementary Figure 2.

Supplementary Figure 3.

Supplementary Figure 4.

Supplementary Figure 5.

Supplementary Figure 6.

Supplementary Figure 7.

Supplementary Figure 8.

Supplementary Figure 9.

Supplementary Figure 10.

Supplementary Figure 11.

Supplementary Figure Legends.

## References

[1] Ioannidis JP (2005). Why most published research findings are false. PLoS Med..

[2] Masouleh SK, Eickhoff SB, Hoffstaedter F, Genon S, Alzheimer's Disease Neuroimaging Initiative (2019). Empirical examination of the replicability of associations between brain structure and psychological variables. Elife.

[3] Lindsay DS (2015). Replication in psychological science. Psychol. Sci..

[4] Pashler H, Wagenmakers E (2012). Editors’ introduction to the special section on replicability in psychological science: A crisis of confidence?. Perspect. Psychol. Sci..

[5] Avinun R, Israel S, Knodt AR, Hariri AR (2020). Little evidence for associations between the big five personality traits and variability in brain gray or white matter. Neuroimage.

[6] Boekel W, Wagenmakers E, Belay L, Verhagen J, Brown S, Forstmann BU (2015). A purely confirmatory replication study of structural brain-behavior correlations. Cortex.

[7] Genon S, Wensing T, Reid A, Hoffstaedter F, Caspers S, Grefkes C (2017). Searching for behavior relating to grey matter volume in a-priori defined right dorsal premotor regions: Lessons learned. Neuroimage.

[8] Shrout PE, Rodgers JL (2018). Psychology, science, and knowledge construction: Broadening perspectives from the replication crisis. Annu. Rev. Psychol..

[9] Botvinik-Nezer R, Holzmeister F, Camerer CF, Dreber A, Huber J, Johannesson M (2020). Variability in the analysis of a single neuroimaging dataset by many teams. Nature.

[10] Simmons JP, Nelson LD, Simonsohn U (2011). False-positive psychology: Undisclosed flexibility in data collection and analysis allows presenting anything as significant. Psychol. Sci..

[11] Carp J (2012). On the plurality of (methodological) worlds: Estimating the analytic flexibility of FMRI experiments. Front. Neurosci..

[12] Martínez K, Madsen SK, Joshi AA, Joshi SH, Roman FJ, Villalon-Reina J (2015). Reproducibility of brain-cognition relationships using three cortical surface-based protocols: An exhaustive analysis based on cortical thickness. Hum. Brain Mapp..

[13] Wagenmakers E, Wetzels R, Borsboom D, van der Maas HLJ, Kievit RA (2012). An agenda for purely confirmatory research. Perspect. Psychol. Sci..

[14] Treiblmaier H, Filzmoser P (2010). Exploratory factor analysis revisited: How robust methods support the detection of hidden multivariate data structures in IS research. Inform. Manag..

[15] Watkins MW (2018). Exploratory factor analysis: A guide to best practice. J. Black Psychol..

[16] Spearman C (1904). "General intelligence," objectively determined and measured. Am. J. Psychol..

[17] Latzman RD, Markon KE (2010). The factor structure and age-related factorial invariance of the Delis–Kaplan Executive Function System (D-KEFS). Assessment.

[18] Friedman NP, Miyake A, Young SE, DeFries JC, Corley RP, Hewitt JK (2008). Individual differences in executive functions are almost entirely genetic in origin. J. Exp. Psychol. Gen..

[19] Collette F, Hogge M, Salmon E, Van der Linden M (2006). Exploration of the neural substrates of executive functioning by functional neuroimaging. Neuroscience.

[20] Armstrong JS, Soelberg P (1968). On the interpretation of factor analysis. Psychol. Bull..

[21] Comrey AL (1978). Common methodological problems in factor analytic studies. J. Consult. Clin. Psychol..

[22] MacCallum R (1983). A comparison of factor analysis programs in SPSS, BMDP, and SAS. Psychometrika.

[23] Weiss, D. J., Rand McNally and Co & United States of America. Multivariate procedures. In *Handbook of Industrial and Organizational Psychology* (ed. Dunnette, M. D.) see ncj-52907 (1976).

[24] Ford JK, MacCallum RC, Tait M (1986). The application of exploratory factor analysis in applied psychology: A critical review and analysis. Pers. Psychol..

[25] Chen J, Patil KR, Weis S, Sim K, Nickl-Jockschat T, Zhou J (2020). Neurobiological divergence of the positive and negative schizophrenia subtypes identified on a new factor structure of psychopathology using non-negative factorization: An international machine learning study. Biol. Psychiatr..

[26] Blackburn R, Renwick SJ, Donnelly JP, Logan C (2004). Big five or big two? superordinate factors in the NEO five factor inventory and the antisocial personality questionnaire. Personal. Individ. Differ..

[27] Amieva H, Phillips L, Della Sala S (2003). Behavioral dysexecutive symptoms in normal aging. Brain Cogn..

[28] Bennett PC, Ong B, Ponsford J (2005). Assessment of executive dysfunction following traumatic brain injury: Comparison of the BADS with other clinical neuropsychological measures. J. Int. Neuropsychol. Soc.: JINS.

[29] Burgess, P. W. Theory and methodology in executive function research. In *Methodology of Frontal and Executive Function* 87–121 (Routledge, 2004).

[30] Chan RC (2001). Dysexecutive symptoms among a non-clinical sample: A study with the use of the dysexecutive questionnaire. Br. J. Psychol..

[31] Robbins TW, James M, Owen AM, Sahakian BJ, Lawrence AD, McInnes L (1998). A study of performance on tests from the CANTAB battery sensitive to frontal lobe dysfunction in a large sample of normal volunteers: Implications for theories of executive functioning and cognitive aging. J. Int. Neuropsychol. Soc..

[32] Bentler PM, Kano Y (1990). On the equivalence of factors and components. Multivar. Behav. Res..

[33] Floyd FJ, Widaman KF (1995). Factor analysis in the development and refinement of clinical assessment instruments. Psychol. Assess..

[34] Gorsuch RL (1990). Common factor analysis versus component analysis: Some well and little known facts. Multivar. Behav. Res..

[35] Costello AB, Osborne J (2005). Best practices in exploratory factor analysis: Four recommendations for getting the most from your analysis. Pract. Assess. Res. Eval..

[36] Arrindell WA, Van der Ende J (1985). An empirical test of the utility of the observations-to-variables ratio in factor and components analysis. Appl. Psychol. Meas..

[37] Guadagnoli E, Velicer WF (1988). Relation of sample size to the stability of component patterns. Psychol. Bull..

[38] Conway JM, Huffcutt AI (2003). A review and evaluation of exploratory factor analysis practices in organizational research. Organ. Res. Methods.

[39] Zelazo, P. D. & Müller, U. Executive function in typical and atypical development. In *Handbook of Childhood Cognitive Development* 445–469 (2002).

[40] Zelazo PD, Carter A, Reznick JS, Frye D (1997). Early development of executive function: A problem-solving framework. Rev. Gen. Psychol..

[41] Lezak MD (1982). The problem of assessing executive functions. Int. J. Psychol..

[42] Miyake A, Friedman NP, Emerson MJ, Witzki AH, Howerter A, Wager TD (2000). The unity and diversity of executive functions and their contributions to complex “frontal lobe” tasks: A latent variable analysis. Cogn. Psychol..

[43] Jurado MB, Rosselli M (2007). The elusive nature of executive functions: A review of our current understanding. Neuropsychol. Rev..

[44] Miyake A, Friedman NP (2012). The nature and organization of individual differences in executive functions: Four general conclusions. Curr. Dir. Psychol. Sci..

[45] Karr JE, Areshenkoff CN, Rast P, Hofer SM, Iverson GL, Garcia-Barrera MA (2018). The unity and diversity of executive functions: A systematic review and re-analysis of latent variable studies. Psychol. Bull..

[46] Delis, D. C., Kaplan, E. & Kramer, J. H. *Delis–Kaplan Executive Function System* (2001).

[47] Floyd RG, Bergeron R, Hamilton G, Parra GR (2010). How do executive functions fit with the Cattell–Horn–Carroll model? Some evidence from a joint factor analysis of the Delis–Kaplan executive function system and the Woodcock–Johnson III tests of cognitive abilities. Psychol. Sch..

[48] McFarland DJ (2020). Factor-analytic evidence for the complexity of the Delis–Kaplan Executive Function System (D-KEFS). Assessment.

[49] Chen J, Müller VI, Dukart J, Hoffstaedter F, Baker JT, Holmes AJ, Vatansever D, Nickl-Jockschat T, Liu X, Derntl B, Kogler L (2020). Intrinsic connectivity patterns of task-defined brain networks allow individual prediction of cognitive symptom dimension of schizophrenia and are linked to molecular architecture. Biol. Psychiatr..

[50] Love J, Selker R, Verhagen J, Marsman M, Gronau QF, Jamil T (2015). Software to sharpen your stats. APS Obs..

[51] Nooner KB, Colcombe S, Tobe R, Mennes M, Benedict M, Moreno A (2012). The NKI-rockland sample: A model for accelerating the pace of discovery science in psychiatry. Front. Neurosci..

[52] Kim J, Mouw KW, Polak P, Braunstein LZ, Kamburov A, Tiao G (2016). Somatic ERCC2 mutations are associated with a distinct genomic signature in urothelial tumors. Nat. Genet..

[53] Hofree M, Shen JP, Carter H, Gross A, Ideker T (2013). Network-based stratification of tumor mutations. Nat. Methods.

[54] Sadanandam A, Lyssiotis CA, Homicsko K, Collisson EA, Gibb WJ, Wullschleger S (2013). A colorectal cancer classification system that associates cellular phenotype and responses to therapy. Nat. Med..

[55] Sotiras A, Resnick SM, Davatzikos C (2015). Finding imaging patterns of structural covariance via non-negative matrix factorization. Neuroimage.

[56] Yang Z, Oja E (2010). Linear and nonlinear projective nonnegative matrix factorization. IEEE Trans. Neural Netw..

[57] Hubert L, Arabie P (1985). Comparing partitions. J. Classif..

[58] Meilă M (2007). Comparing clusterings—An information based distance. J. Multivar. Anal..

[59] Raguideau S, Plancade S, Pons N, Leclerc M, Laroche B (2016). Inferring aggregated functional traits from metagenomic data using constrained non-negative matrix factorization: Application to fiber degradation in the human gut microbiota. PLoS Comput. Biol..

[60] Fisk JE, Sharp CA (2004). Age-related impairment in executive functioning: Updating, inhibition, shifting, and access. J. Clin. Exp. Neuropsychol..

[61] Lehto JE, Juujärvi P, Kooistra L, Pulkkinen L (2003). Dimensions of executive functioning: Evidence from children. Br. J. Dev. Psychol..

[62] Hull R, Martin RC, Beier ME, Lane D, Hamilton AC (2008). Executive function in older adults: A structural equation modeling approach. Neuropsychology.

[63] Cattell RB (1966). The scree test for the number of factors. Multivar. Behav. Res..

[64] Horn JL (1965). A rationale and test for the number of factors in factor analysis. Psychometrika.

[65] D'agostino, R. B. & Russell, H. K. Scree test. In *Encyclopedia of Biostatistics*, Vol. 7 (2005).

[66] Wood ND, Akloubou Gnonhosou DC, Bowling JW (2015). Combining parallel and exploratory factor analysis in identifying relationship scales in secondary data. Marriage Fam. Rev..

[67] Franklin SB, Gibson DJ, Robertson PA, Pohlmann JT, Fralish JS (1995). Parallel analysis: A method for determining significant principal components. J. Veg. Sci..

[68] McDonald RP, Marsh HW (1990). Choosing a multivariate model: Noncentrality and goodness of fit. Psychol. Bull..

[69] The jamovi Project. *jamovi *(Version 1.2) [Computer Software] (2021). Retrieved from https://www.jamovi.org.

[70] Deckel AW, Hesselbrock V (1996). Behavioral and cognitive measurements predict scores on the MAST: A 3-year prospective study. Alcohol.: Clin. Exp. Res..

[71] Testa R, Bennett P, Ponsford J (2012). Factor analysis of nineteen executive function tests in a healthy adult population. Arch. Clin. Neuropsychol..

[72] Vaughan L, Giovanello K (2010). Executive function in daily life: Age-related influences of executive processes on instrumental activities of daily living. Psychol. Aging.

[73] Baron SI (2004). Delis–Kaplan executive function system. Child Neuropsychol..

[74] Karr JE, Hofer SM, Iverson GL, Garcia-Barrera MA (2019). Examining the latent structure of the Delis–Kaplan executive function system. Arch. Clin. Neuropsychol..

[75] Brydges CR, Reid CL, Fox AM, Anderson M (2012). A unitary executive function predicts intelligence in children. Intelligence.

[76] Hughes C, Ensor R (2011). Individual differences in growth in executive function across the transition to school predict externalizing and internalizing behaviors and self-perceived academic success at 6 years of age. J. Exp. Child Psychol..

[77] Fournier-Vicente S, Larigauderie P, Gaonac’h D (2008). More dissociations and interactions within central executive functioning: A comprehensive latent-variable analysis. Acta Physiol. (Oxf.).

[78] de Frias CM, Dixon RA, Strauss E (2006). Structure of four executive functioning tests in healthy older adults. Neuropsychology.

[79] Glisky EL, Alexander GE, Hou M, Kawa K, Woolverton CB, Zigman EK (2020). Differences between young and older adults in unity and diversity of executive functions. Aging Neuropsychol. Cogn..

[80] Traykov L, Raoux N, Latour F, Gallo L, Hanon O, Baudic S (2007). Executive functions deficit in mild cognitive impairment. Cogn. Behav. Neurol..

[81] Zhang Y, Han B, Verhaeghen P, Nilsson L (2007). Executive functioning in older adults with mild cognitive impairment: MCI has effects on planning, but not on inhibition. Aging Neuropsychol. Cogn..

[82] Brandt J, Aretouli E, Neijstrom E, Samek J, Manning K, Albert MS (2009). Selectivity of executive function deficits in mild cognitive impairment. Neuropsychology.

[83] Elliott R (2003). Executive functions and their disorders: Imaging in clinical neuroscience. Br. Med. Bull..

[84] Badre D, Nee DE (2018). Frontal cortex and the hierarchical control of behavior. Trends Cogn. Sci..

[85] Berg EA (1948). A simple objective technique for measuring flexibility in thinking. J. Gen. Psychol..

