# Supplementary material for: A machine learning approach for the factorization of psychometric data with application to the Delis Kaplan Executive Function System
Source: Sci Rep. 2021 Aug 19;11:16896. doi: 10.1038/s41598-021-96342-3 (PMC8377093; doi:10.1038/s41598-021-96342-3)
Supplement: Supplementary file 1 — Supplementary Figure 1. [file 41598_2021_96342_MOESM1_ESM.pdf]

Education and Occupation of Participants

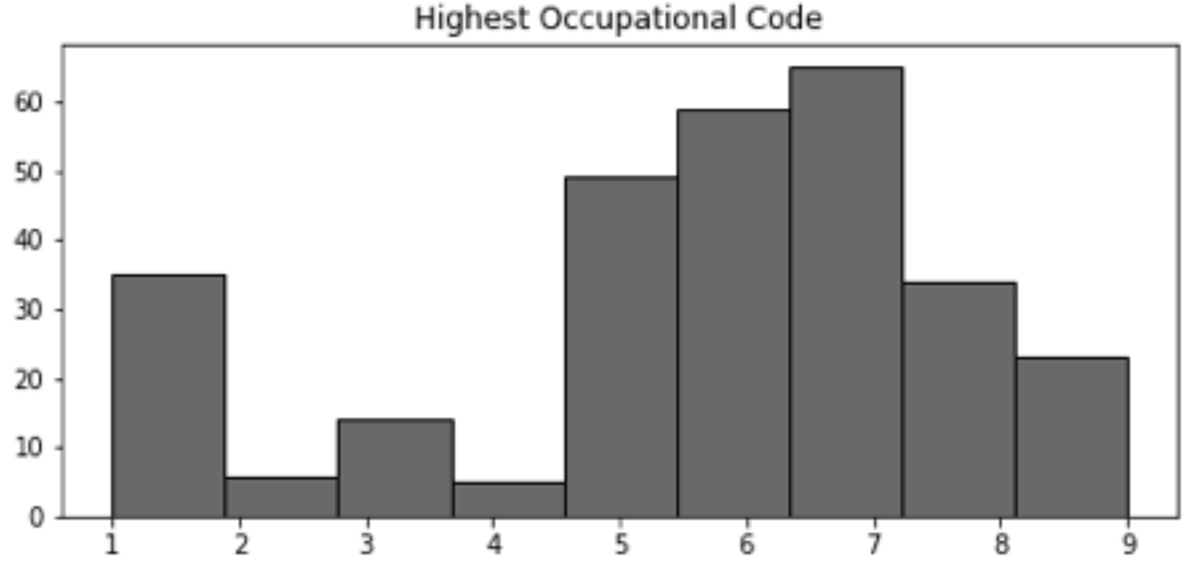

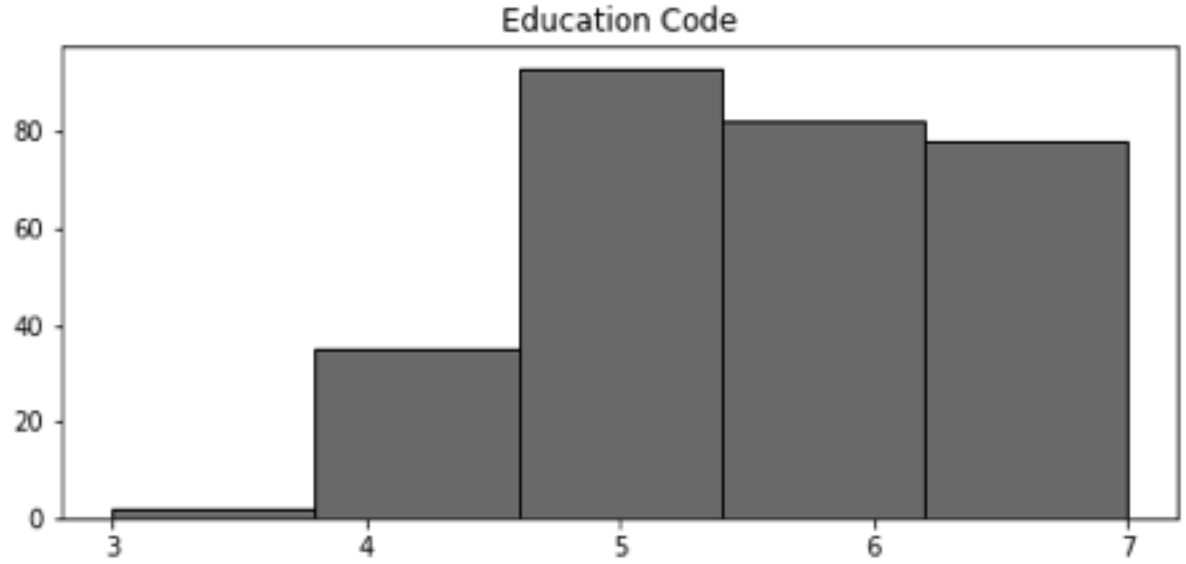

**Highest Occupational Code**
9-Higher executive; proprietor of large businesses, major professional
8-Administrators, lesser professionals, proprietor of medium-sized business
7-Smaller business owners, farm owners, managers, minor professionals
6-Technicians, semi-professionals, small business owners (business valued at $50,000-70,000)
5-Clerical and sales workers, small farm and business owners (business valued at $25,000-50,000)
4-Smaller business owners (< $25,000), skilled manual laborers, craftsman, tenant farmers
3-Machine operators and semi-skilled workers
2-Unskilled workers
1-Farm laborers, menial service workers, students, housewives (dependent on welfare, no regular occupation)
**Education Code**
7-Graduate/professional training (graduate degree)
6-Standard college or university graduation
5-Partial college (at least one year or specialized training)
4-High school graduate
3-Partial high school (10th or 11th grade)
2-Junior high school (including 9th grade)
1-Less than seventh grade
