# Supplementary figures and images for: A machine learning approach for the factorization of psychometric data with application to the Delis Kaplan Executive Function System
Source: Sci Rep. 2021 Aug 19;11:16896. doi: 10.1038/s41598-021-96342-3 (PMC8377093; doi:10.1038/s41598-021-96342-3)

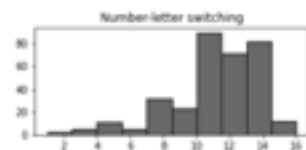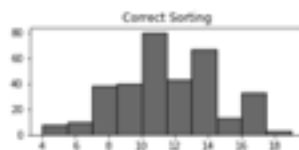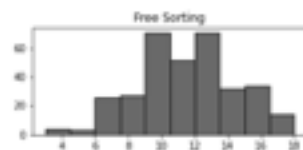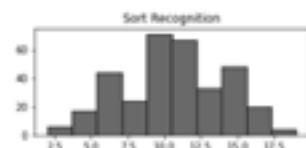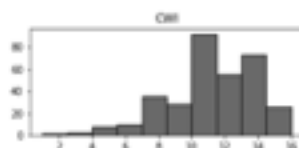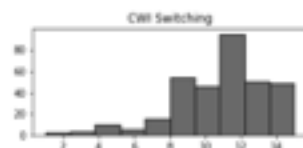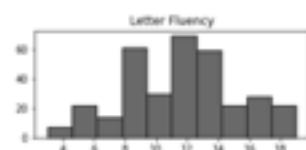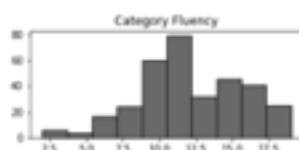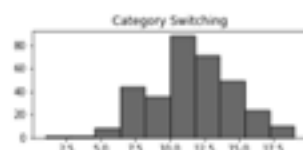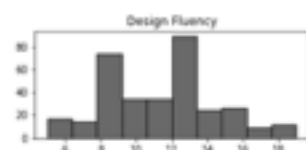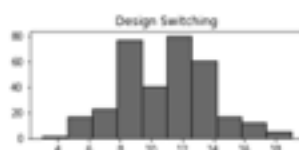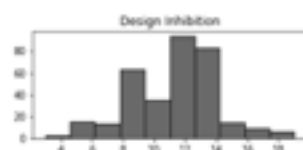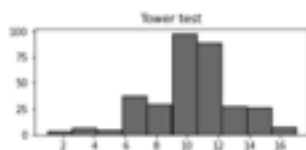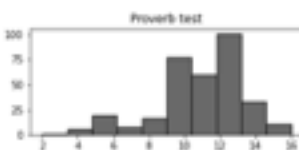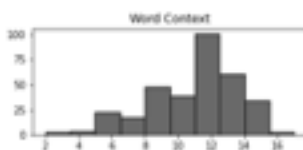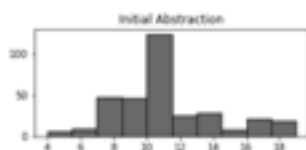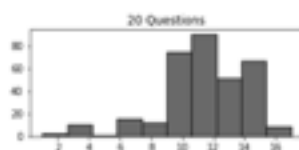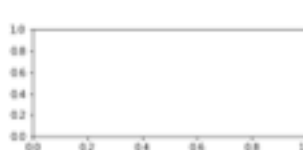

Supplement: Supplementary file 2 — Supplementary Figure 2. [file 41598_2021_96342_MOESM2_ESM.pdf]

# Factor structure for subsample of males

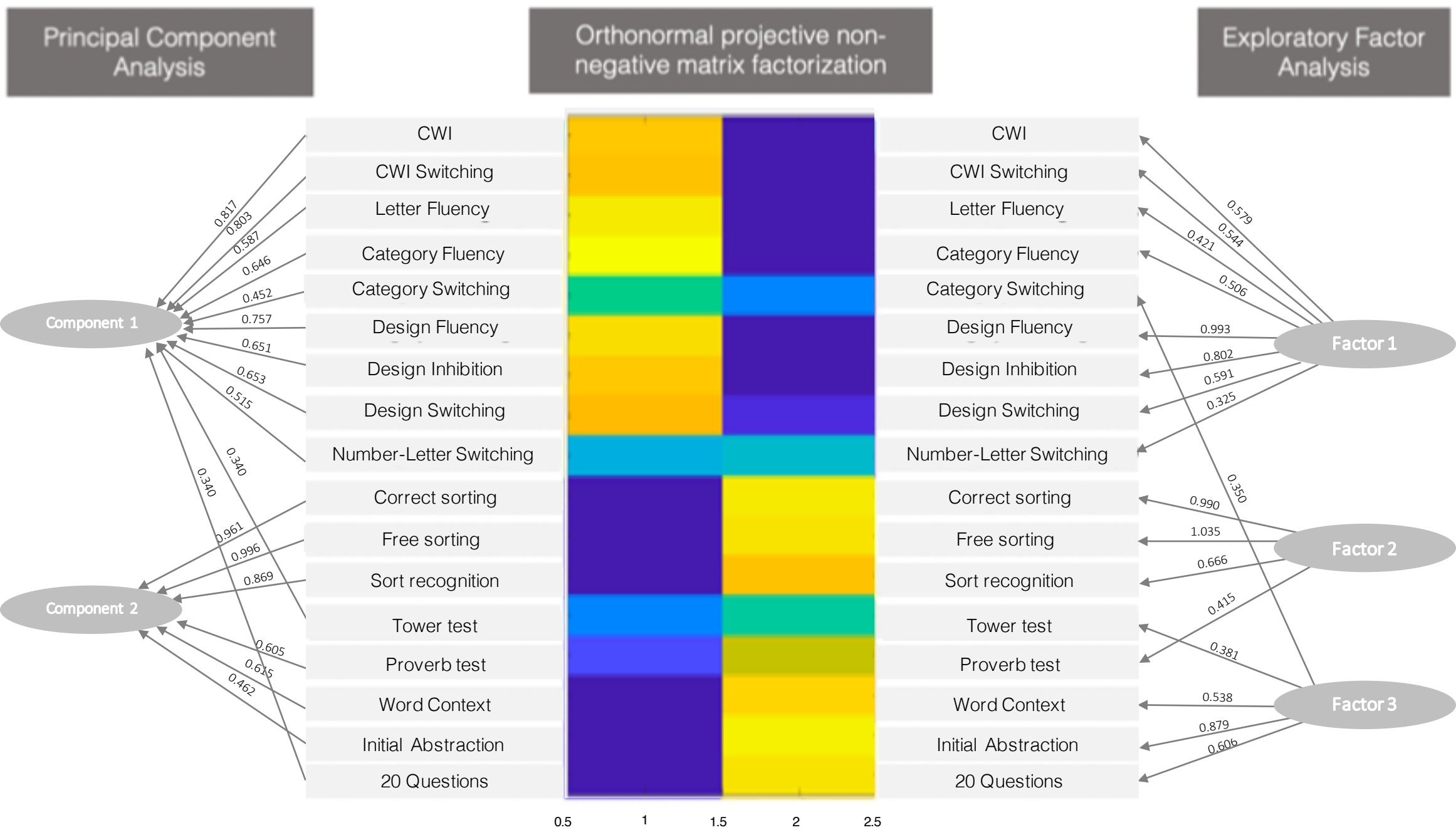

Supplement: Supplementary file 5 — Supplementary Figure 5. [file 41598_2021_96342_MOESM5_ESM.pdf]

Factor structure for subsample of younger adults

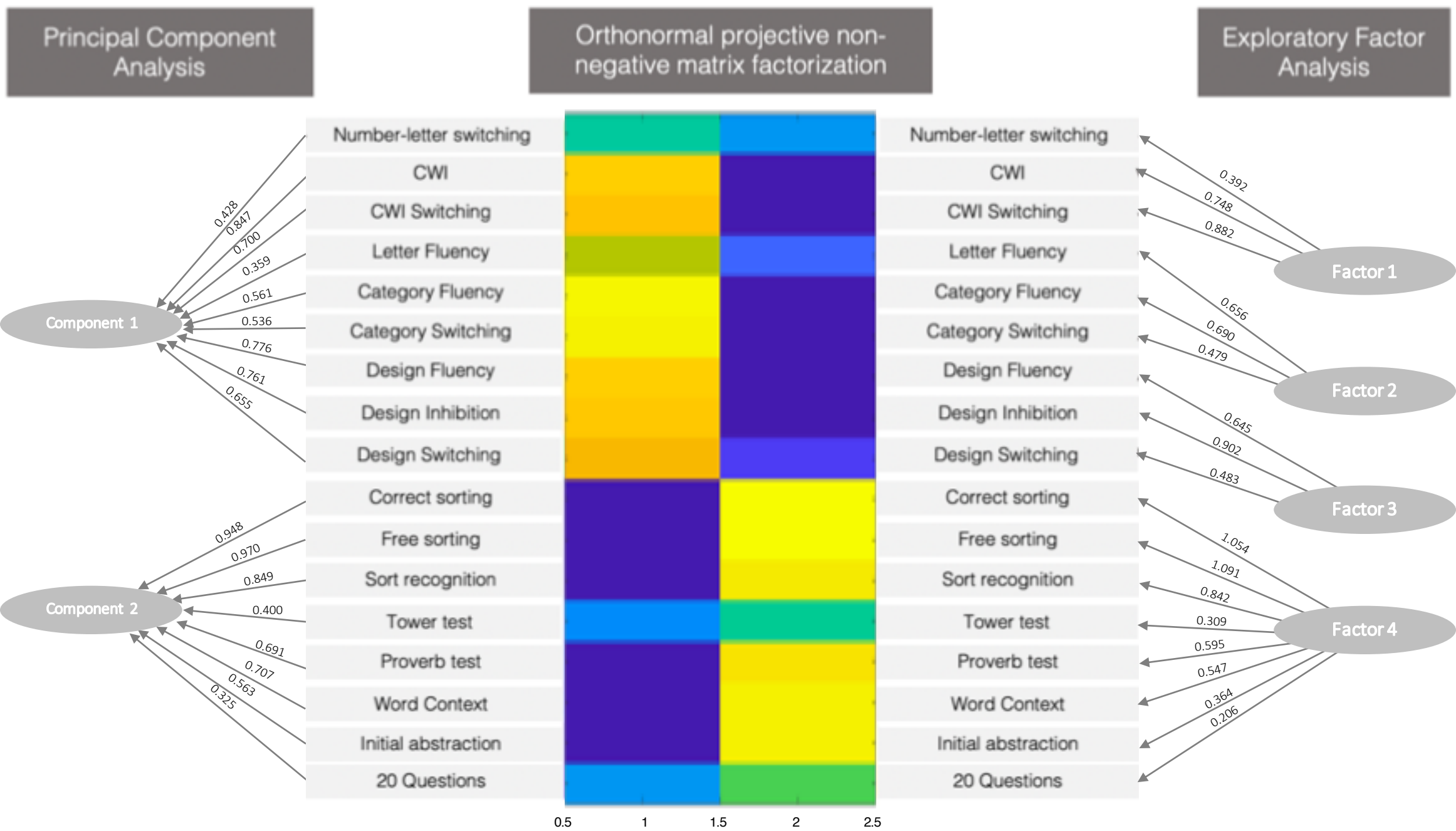

Supplement: Supplementary file 11 — Supplementary Figure 11. [file 41598_2021_96342_MOESM11_ESM.pdf]
