# Supplementary material for: A machine learning approach for the factorization of psychometric data with application to the Delis Kaplan Executive Function System
Source: Sci Rep. 2021 Aug 19;11:16896. doi: 10.1038/s41598-021-96342-3 (PMC8377093; doi:10.1038/s41598-021-96342-3)
Supplement: Supplementary file 3 — Supplementary Figure 3. [file 41598_2021_96342_MOESM3_ESM.pdf]

Normality Testing Results

Variable	D'Agostino's K^2		Shapiro-Wilk	
	Statistic	p-value	Statistic	p-value
Number-letter Switching	55.599	0.000	0.921	0.000
Correct Sorting	1.893	0.388	0.985	0.001
Free Sort	2.917	0.233	0.986	0.002
Sort Recognition	5.133	0.077	0.986	0.002
CWI	28.199	0.000	0.959	0.000
CWI Switching	39.532	0.000	0.940	0.000
Letter Fluency	6.987	0.030	0.984	0.001
Category Fluency	1.208	0.547	0.982	0.000
Category Switching	0.865	0.649	0.982	0.000
Design Fluency	5.626	0.060	0.976	0.000
Design Switching	3.627	0.163	0.983	0.001
Design Inhibition	0.687	0.709	0.982	0.000
Tower Test	7.734	0.021	0.980	0.000
Proverb Test	31.590	0.000	0.942	0.000
Word Context	20.971	0.000	0.953	0.000
Initial Abstraction	29.904	0.000	0.921	0.000
20 Questions	45.501	0.000	0.942	0.000
