# Supplementary material for: A machine learning approach for the factorization of psychometric data with application to the Delis Kaplan Executive Function System
Source: Sci Rep. 2021 Aug 19;11:16896. doi: 10.1038/s41598-021-96342-3 (PMC8377093; doi:10.1038/s41598-021-96342-3)

Factor structure for subsample of females

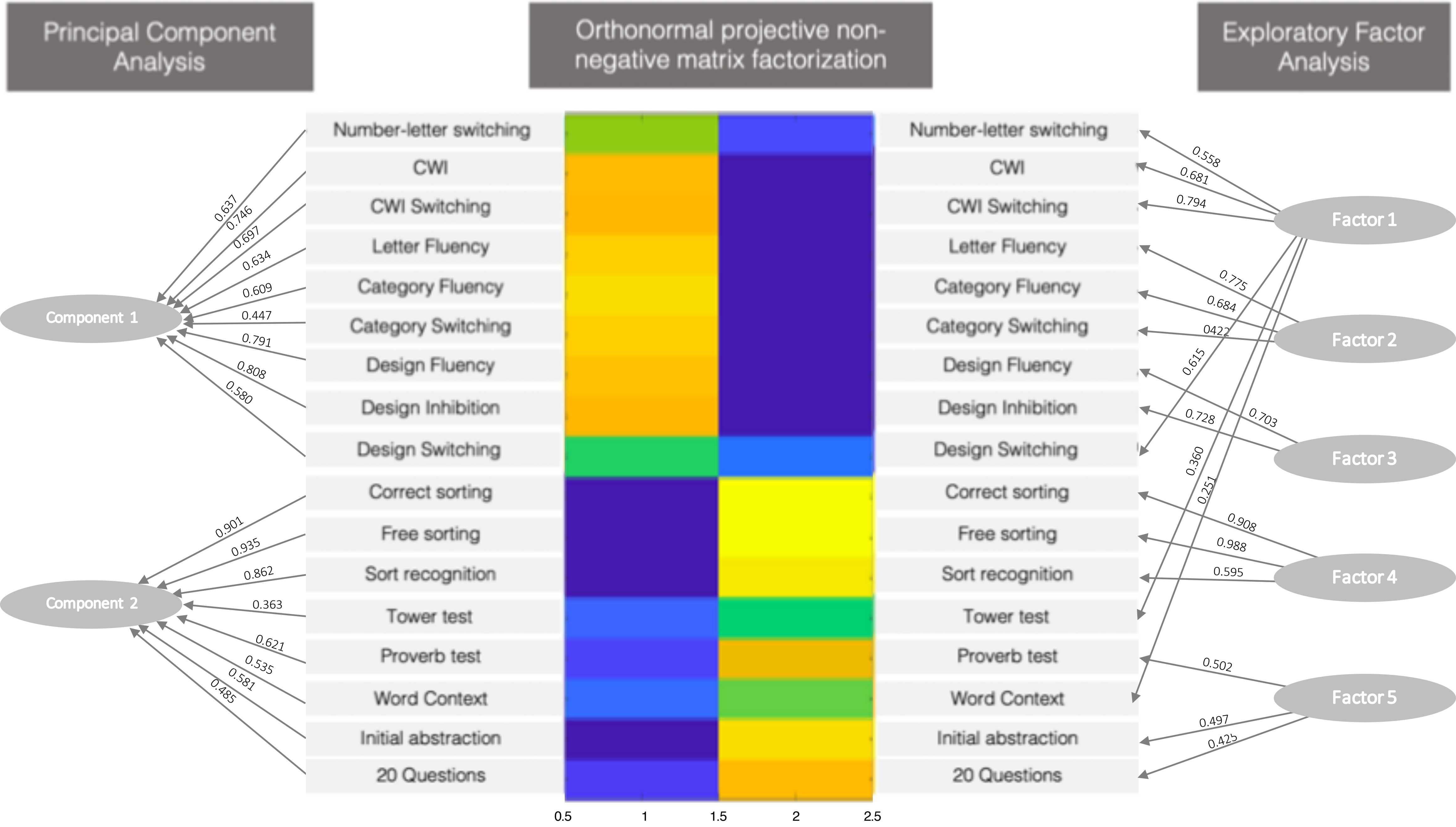

Supplement: Supplementary file 7 — Supplementary Figure 7. [file 41598_2021_96342_MOESM7_ESM.pdf]
