# Supplementary material for: A machine learning approach for the factorization of psychometric data with application to the Delis Kaplan Executive Function System
Source: Sci Rep. 2021 Aug 19;11:16896. doi: 10.1038/s41598-021-96342-3 (PMC8377093; doi:10.1038/s41598-021-96342-3)
Supplement: Supplementary file 12 — Supplementary Figure Legends. [file 41598_2021_96342_MOESM12_ESM.docx]

**Supplementary Figure 1**: Education and Occupation of Participants

Frequency distributions depicting the education and occupation of the participants

**Supplementary Figure 2**: Frequency Distributions

Frequency distributions for each of the 17 variables used in the analyses.

**Supplementary Figure 3**: Normality Testing Results

Normality testing results based on Shapiro-Wilk and D’Agostino’s K^2^ tests

**Supplementary Figure 4**: Stability measures for subsample of males

Stability measures for males

**Supplementary Figure 5**: Factor structure for subsample of males

Factor structure and factor loadings resulting from the PCA, EFA and OPNMF analyses for males

**Supplementary Figure 6**: Stability measures for subsample of females

Stability measures for females

**Supplementary Figure 7**: Factor structure for subsample of females

Factor structure and factor loadings resulting from the PCA, EFA and OPNMF analyses for females

**Supplementary Figure 8**: Stability measures for subsample of older adults

Stability measures for older adults

**Supplementary Figure 9**: Factor structure for subsample of older adults

Factor structure and factor loadings resulting from the PCA, EFA and OPNMF analyses for older adults

**Supplementary Figure 10**:Stability measures for subsample of younger adults

Stability measures for younger adults

**Supplementary Figure 11**:Factor structure for subsample of younger adults

Factor structure and factor loadings resulting from the PCA, EFA and OPNMF analyses for younger adults
